# Optical control of insulin release using a photoswitchable sulfonylurea

**DOI:** 10.1038/ncomms6116

**Published:** 2014-10-14

**Authors:** Johannes Broichhagen, Matthias Schönberger, Simon C. Cork, James A. Frank, Piero Marchetti, Marco Bugliani, A. M. James Shapiro, Stefan Trapp, Guy A. Rutter, David J. Hodson, Dirk Trauner

**Affiliations:** 1 Department of Chemistry, Ludwig-Maximilians-Universität München, and Munich Center for Integrated Protein Science, Butenandtstrasse 5-13, 81377 München, Germany; 2 Division of Biosciences, Department of Neuroscience, Physiology and Pharmacology, University College London, London WC1E 6BT, UK; 3 Department of Clinical and Experimental Medicine, Islet Cell Laboratory, University of Pisa, 56126 Pisa, Italy; 4 Clinical Islet Laboratory and Clinical Islet transplant program, University of Alberta, Edmonton, Alberta T6G 2C8, Canada; 5 Section of Cell Biology, Department of Medicine, Imperial College London, Imperial Centre for Translational and Experimental Medicine, Hammersmith Hospital, Du Cane Road, London W12 0NN, UK

## Abstract

Sulfonylureas are widely prescribed for the treatment of type 2 diabetes mellitus (T2DM). Through their actions on ATP-sensitive potassium (K_ATP_) channels, sulfonylureas boost insulin release from the pancreatic beta cell mass to restore glucose homeostasis. A limitation of these compounds is the elevated risk of developing hypoglycemia and cardiovascular disease, both potentially fatal complications. Here, we describe the design and development of a photoswitchable sulfonylurea, JB253, which reversibly and repeatedly blocks K_ATP_ channel activity following exposure to violet-blue light. Using *in situ* imaging and hormone assays, we further show that JB253 bestows light sensitivity upon rodent and human pancreatic beta cell function. Thus, JB253 enables the optical control of insulin release and may offer a valuable research tool for the interrogation of K_ATP_ channel function in health and T2DM.

## Abstract (web summary)

Sulfonylureas are widely used anti-diabetic drugs, which promote insulin release by blocking a pancreatic ion channel. Here the authors create a photoswitchable sulfonylurea derivative and use it to control insulin release from cultured cells and isolated pancreatic islets by illumination with blue light.

## Graphical abstract

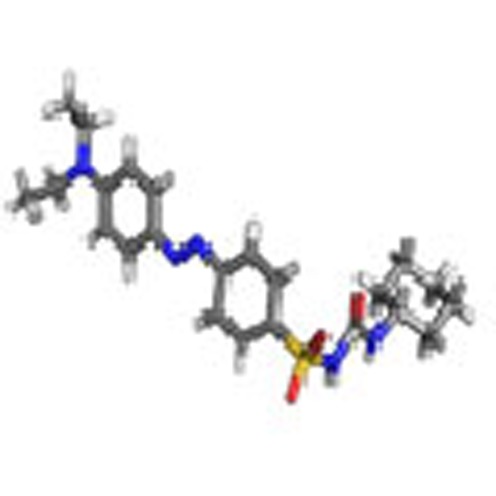

Type 2 diabetes mellitus (T2DM) is a global health-care epidemic associated with life-changing sequelae ranging from blindness to cancer^1^,^2^. This endocrine disease, which currently affects 1 in 12 of the adult population worldwide, involves a disturbance of normal glucose homeostasis due to failure of the pancreatic beta cell mass to adequately compensate for increased peripheral insulin resistance^3^. As such, the rescue of insulin release through the coaxing of beta cell activity remains a therapeutically desirable approach for the long-term restoration of normal glucose levels.

Sulfonylureas, which target ATP-sensitive potassium (K^+^) (K_ATP_) channels, are a mainstay of diabetes therapy^4^,^5^,^6^. K_ATP_ channels are hetero-octameric structures composed of four regulatory sulfonylurea receptor subunits (SUR1) and four Kir6.2 subunits, the latter forming a central ion pore that permits K^+^ efflux^7^,^8^,^9^. By binding to SUR1, sulfonylureas block the Kir6.2 inward rectifier, leading to cell depolarization and opening of voltage-dependent Ca^2+^ channels (VDCC)^10^,^11^. The ensuing Ca^2+^ influx^12^,^13^, along with K_ATP_ channel-independent signals^14^, drives various downstream processes that ultimately converge on the exocytosis of insulin^15^. Elevated circulating insulin can then act on target tissues to improve glucose uptake, hepatic glycogenesis and fatty acid synthesis^16^ (Supplementary Fig. 1).

While sulfonylureas are widely prescribed because of their effectiveness and relative inexpensiveness, they have a range of off-target effects that limits their therapeutic use. For example, sulfonylureas can provoke prolonged episodes of low blood glucose due to hyperinsulinemia^17^, elevate cardiovascular disease risk^18^ and induce weight gain^19^. Conversely, there is a lack of tools for the precise functional dissection of K_ATP_ channels located not only in the pancreas, but also in the brain^20^,^21^, heart^22^ and vascular smooth muscle^23^. With this in mind, we set out to combine the glucose-lowering attributes of sulfonylureas with the exquisite spatiotemporal control conferred by possession of photoresponsive elements^24^,^25^.

Here, we showcase JB253, a ‘fourth-generation’ sulfonylurea based on glimepiride that bears an azobenzene photoswitch, endowing K_ATP_ channels with remarkable photocontrollable properties (Fig. 1a). We demonstrate that JB253 offers sensitive, reversible and repeated manipulation of K_ATP_ channel state and beta cell activity with visible light, yielding optical control over insulin release. Thus, JB253 may allow the selective targeting of K_ATP_ channels in the pancreas and elsewhere.

## Results

### Design and synthesis of JB253

**Figure 1:**
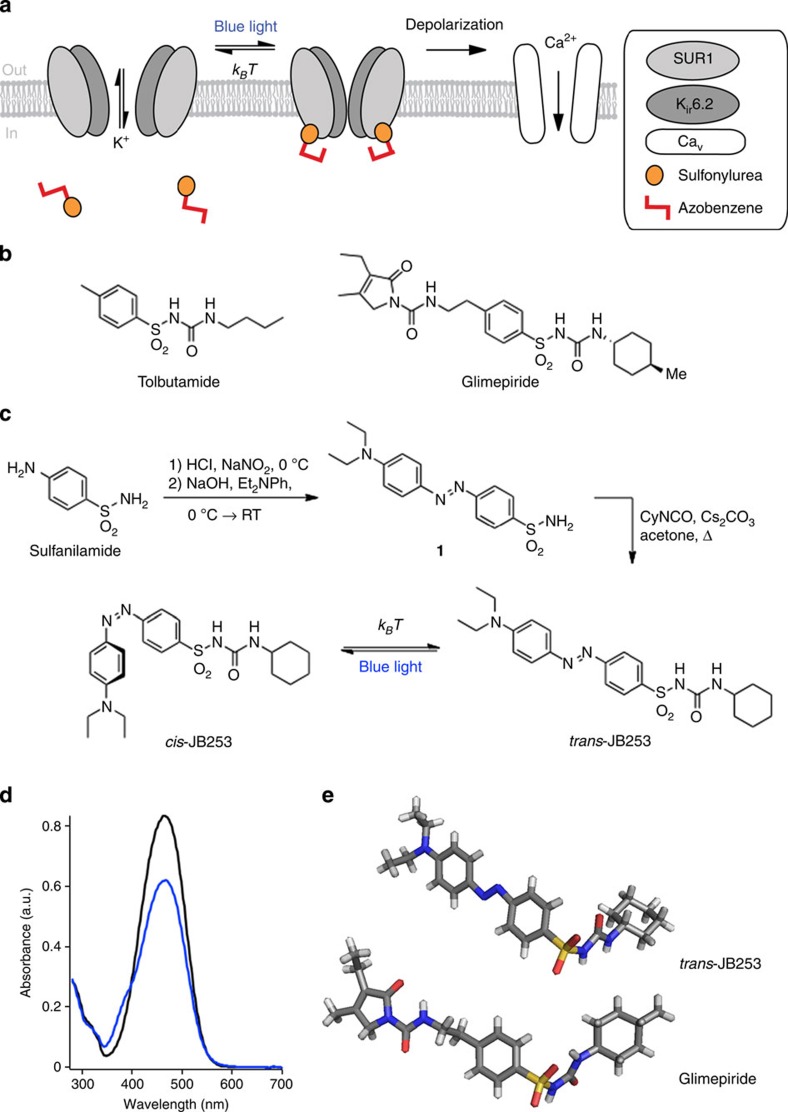
Photopharmacology of K_ATP_ channels: design, synthesis and characteristics of JB253. (**a**) The logic of a photoswitchable sulfonylurea: upon photoisomerization to the *cis*-state, JB253 becomes more active, closing the K_ATP_ channel. Thermal relaxation makes the compound less active or leads to dissociation, restoring the open form of the channel. Closure of K_ATP_ channels leads to depolarization, promoting calcium influx and ultimately insulin release. (**b**) Chemical structure of tolbutamide and glimepiride, which served as templates for JB253. (**c**) Synthesis, structure and switching characteristics of JB253. Sulfanilamide undergoes diazotization and is trapped with *N*,*N*-diethylaniline to yield an azobenzene-sulfonamide, which is converted to JB253 by cyclohexyl isocyanate. trans-JB253 can be reversibly switched to cis-JB253 with blue light and relaxes thermally. (**d**) ultraviolet/Vis spectra of JB253 in the dark (black) and during constant illumination with 460 nm (blue). (**e**) Crystal structure of trans-JB253 (CCDC: 1014606) and glimepiride (CSD: TOHBUN01) showing the structural similarity of both sulfonylureas.

Distinct substitution patterns are found within different classes of arylsulfonylurea drugs: while there may be a variety of moieties on the aryl-ring, ranging from a simple methyl group in tolbutamide to more complex structures such as a linked pyrrolidinone in glimepiride (Fig. 1b), the terminal nitrogen in sulfonylureas is usually substituted with an aliphatic group. We reasoned that, to generate a photoswitchable analogue, the aromatic core of the sulfonylurea drugs could be extended to an azobenzene. Furthermore, we aimed for a cyclohexyl substituent on the urea moiety that mimics the corresponding substituent on glimepiride. Using a simple three-step procedure commencing with sulfanilamide, *N*,*N*-diethylaniline and cyclohexyl isocyanate, JB253 could be synthesized rapidly and inexpensively in large quantities via the sulfonamide-azobenzene (*E*)-4-((4-(diethylamino)phenyl)-diazenyl)benzenesulfonamide (Fig. 1c; Supplementary Figs 2–4). Initial photochromic characteristics of JB253 were measured using a ultraviolet/visible (Vis) spectrophotometer equipped with a monochromator, affording a single broad band as expected for a push-pull-azobenzene-system (*λ*_max_=472 nm) (Fig. 1d).

Azobenzenes are known to be photoconverted between their *cis*- and *trans*-state by excitation with different wavelengths of light, or alternatively by illumination and dark-relaxation. Indeed, JB253 was readily converted to its *cis*-state by applying wavelengths ranging from *λ*=400 to 500 nm (peak *λ*=472 nm), while thermal relaxation to its *trans*-state occurred rapidly in the dark. X-ray diffractometry revealed a high degree of structural similarity between trans-JB253 and glimepiride crystals^26^ (Fig. 1e) (see Supplementary Table 1). Whereas glimepiride in solution rotates freely around its ethylene carbon chain to adopt various possible binding conformations, JB253 is rigid unless illuminated and so can only adopt two conformations depending on isomeric state (that is, *trans*- or *cis*-).

Attaching lipophilic azobenzene units normally renders molecules poorly soluble in water and aqueous buffers, an obvious drawback for their use in biological systems. JB253, however, demonstrates excellent water solubility (≥0.1 mM) when diluted from a 50 mM stock solution in dimethyl sulfoxide (DMSO), presumably due to its acidity (*pK*_*a*_ (trans-JB253)=4.76; see Supplementary Fig. 5). These features were a promising entry point for our subsequent studies using mammalian tissue.

### JB253-binding studies

**Figure 2:**
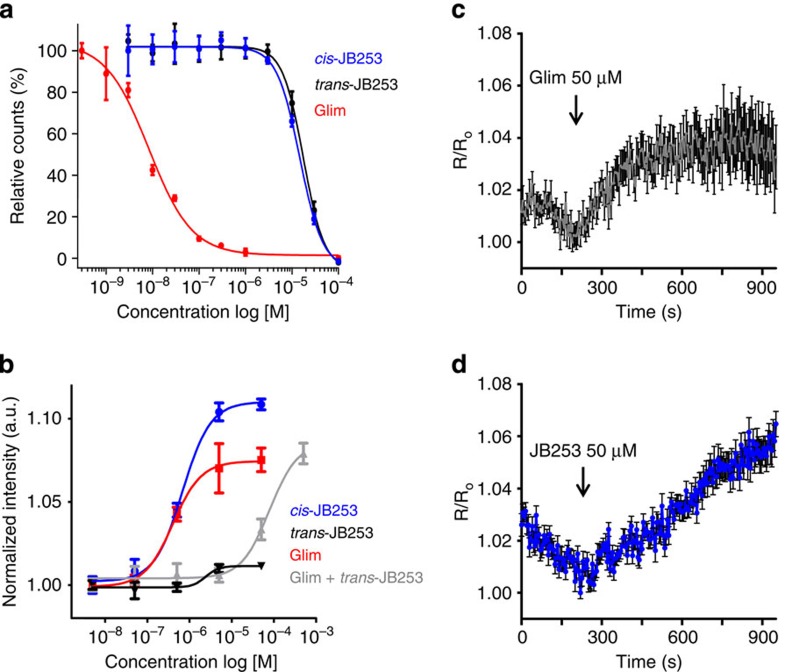
JB253 binding and concentration–response studies. (**a**) Binding affinity of glimepiride (Glim) (red, IC_50_=8.3 nM), trans-JB253 (black, IC_50_=17.6 μM) and cis-JB253 (blue, IC_50_=14.8 μM) were indirectly determined in SUR1-expressing HEK293t cells using displacement of [3H]-glibenclamide (data points fitted using the Hill equation) (*n*=3 independent repeats). Note that, due to the potential for thermal dark-relaxation during the harvesting and washing steps, effects of photoswitching on JB253 binding affinity could not be excluded. Values represent the mean±s.d. (**b**) cis-JB253 and glimepiride (Glim) possess similar concentration–response curves for the stimulation of [Ca^2+^]_i_ in mouse islets, while trans-JB253 is largely ineffective. The concentration–response for glimepiride is right-shifted in the presence of a saturating concentration (100 μM) of trans-JB253 (data points fitted using nonlinear regression) (*n*=3 recordings). (**c**) HEK293t cells expressing a full-length Epac2-camps probe respond to glimepiride with decreases in Förster resonance energy transfer (FRET) (represented here as an increase in *R*/*R*_o_) (*n*=28 cells from 4 recordings). (**d**) As in **c**, but cis-JB253. Values represent the mean±s.e.m. for (**b**–**d**).

To determine the binding affinity of JB253 to SUR1 relative to a known sulfonylurea (that is, glimepiride), [3H]-glibenclamide displacement assays were performed. JB253 bound SUR1 with a 1,000-fold lower affinity compared with glimepiride, and this was unaffected by illumination (half-maximal inhibitory concentration (IC_50_)=8.3 nM versus 17.6 μM versus 14.8 μM for glimepiride versus trans-JB253 versus cis-JB253, respectively) (Fig. 2a). However, owing to the potential for rapid thermal dark-relaxation during the wash cycles (see below), we were unable to exclude a role for *trans*- to *cis*- isomerization in strengthening JB253 binding affinity. Therefore, to compare the activity profiles of trans-JB253, cis-JB253 and glimepiride using a functionally relevant readout, concentration–response experiments were conducted in mouse islets. The effector concentration for half-maximum response (EC_50_) of cis-JB253 for cytosolic Ca^2+^ rises was found to be 675 nM, similar to that obtained for glimepiride in the same system (EC_50_ glimepiride=399 nM) (Fig. 2b). The concentration–response curve for glimepiride was right-shifted in the presence of a saturating concentration of trans-JB253, demonstrating the presence of competitive agonism even under dark conditions (Fig. 2b).

Since most sulfonlyureas have been reported to bind and activate Exchange Protein directly Activated by cAMP 2A (Epac2A)^27^, an important mediator of insulin secretion^28^,^29^, the presence of interactions with JB253 was assessed using a Förster resonance energy transfer (FRET)-based approach. To enable this, a full-length Epac2-camps biosensor containing the sulfonylurea-binding site was encoded in HEK293t cells^29^. Confirming the existence of sufonylurea–Epac2A interactions, application of either glimepiride (Fig. 2c) or cis-JB253 (Fig. 2d) decreased FRET to a similar extent (Δ*R*/*R*_o_=0.052 versus 0.064 a.u., glimepiride versus JB253, respectively; NS, not significant, Student’s *t*-test).

### JB253 allows photoswitching of K_ATP_ channels

**Figure 3:**
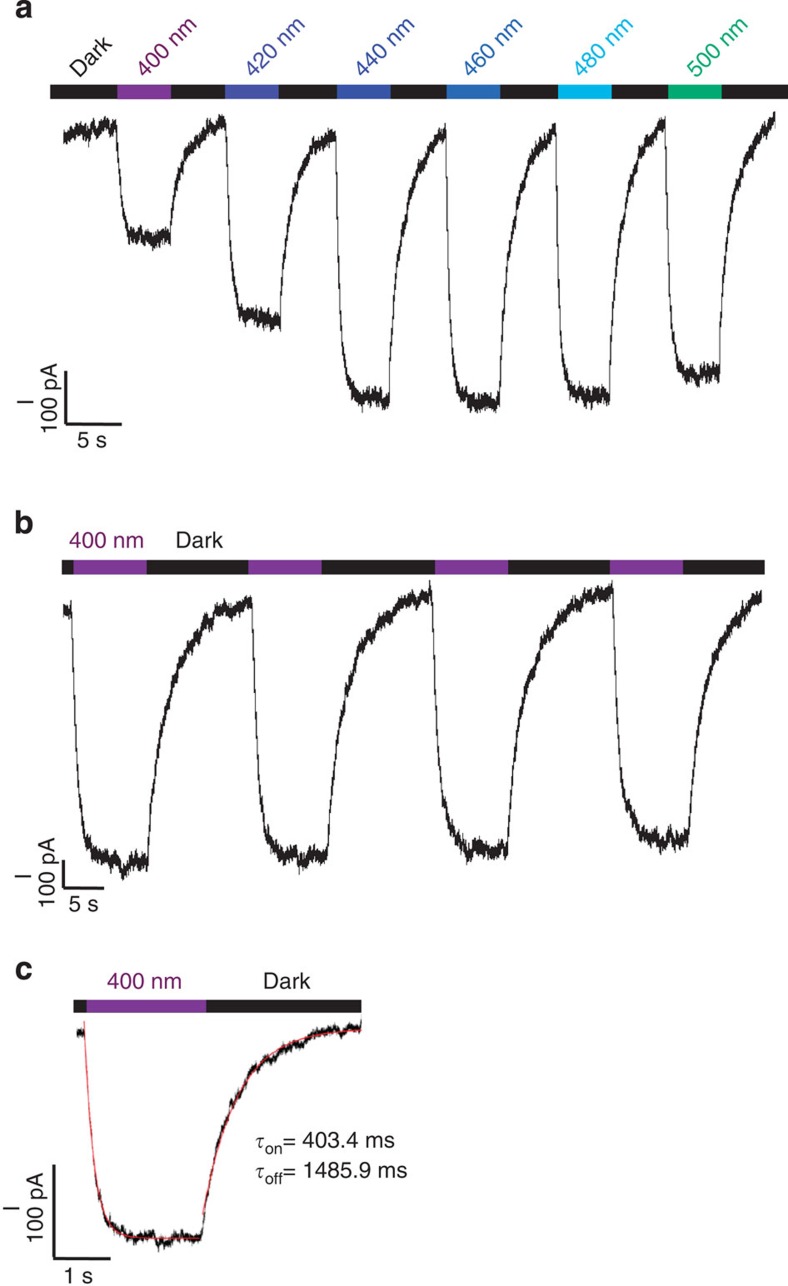
JB253 confers photoswitching on K_ATP_ channels. (**a**) Photocurrents recorded from HEK293t cells transfected with plasmids encoding Kir6.2 and SUR1 using the whole-cell patch-clamp configuration (holding potential −60 mV). JB253-treated cells respond to wavelengths between 400 and 500 nm with a reduction in the magnitude of the K^+^ inward rectifier current. (**b**) JB253 allows reversible and repeated closure of K_ATP_ channels in response to light–dark cycles using *λ*=400 nm to induce *cis*-isomerization (purple) and relaxation in the dark (black). (**c**) Kinetics of light-triggered block and thermal restoration of K_ATP_ channel currents mediated by JB253. In all cases, traces represent *n*=4 cells.

We sought first to investigate whether JB253 could yield optical control over K_ATP_ channel activity using a system free from confounding effects of glucose metabolism. To enable this, K_ATP_ channels were heterologously expressed in HEK293t cells by transfection with plasmids encoding the Kir6.2 and SUR1 subunits along with green fluorescent protein. Tolbutamide and diazoxide-sensitive inward-rectifying K^+^ currents could be recorded in transfected cells, confirming the functional assembly of K_ATP_ channels. In the dark state, JB253 partly reduced K^+^ current amplitude within a few seconds. This was, however, a fraction of that observed during 500 μM tolbutamide application (Supplementary Fig. 6a,b). Subsequent illumination of JB253 with wavelengths between 400 and 500 nm further closed the channel (Fig. 3a), with ~45–72% block being achieved relative to that recorded using 500 μM tolbutamide (Supplementary Table 2). The reversal potential was close to the expected equilibrium potential for K^+^, and this was unaffected by molecule orientation (−90.0±1.8 versus −87.8±1.6 mV, dark versus illuminated; not significant) (Supplementary Fig. 6c,d). As such, JB253 possesses the advantageous property of becoming a high-affinity K_ATP_ channel blocker upon illumination.

Using a wavelength of 400 nm, heterologously expressed K_ATP_ channels could be repeatedly opened and closed in JB253-treated preparations without obvious desensitization (difference in ΔI [pA] between first and last switch=14.9±7.6%) (Fig. 3b; Supplementary Tables 3 and 4). Upon exposure to 400 nm, rapid block was observed (*τ*_on_=0.4 s) (Fig. 3c). When the light source was shut off, thermal relaxation was fast, returning the K_ATP_ channel to baseline levels within a couple of seconds (*τ*_off_=1.5 s) (Fig. 3c; Supplementary Tables 3 and 4). While maximal photoblock of K_ATP_ channels was observed at 460 nm, significant effects were also obtained with violet light (that is, 405 nm), which was more compatible with fluorescence imaging of pancreatic beta cell function (see below). Confirming that JB253 could photoswitch endogenous K_ATP_ channels, hyperpolarizing currents were reversibly blocked in MIN6 beta cells following illumination (Supplementary Fig. 7).

### Functional interrogation of beta cells within mouse islets

Stimulus–secretion coupling in beta cells relies on the closure of K_ATP_ channels, Ca^2+^ influx through VDCC and release of insulin granules^12^. We therefore attempted to manipulate beta cell activity by optically controlling K_ATP_ channels with JB253.

**Figure 4:**
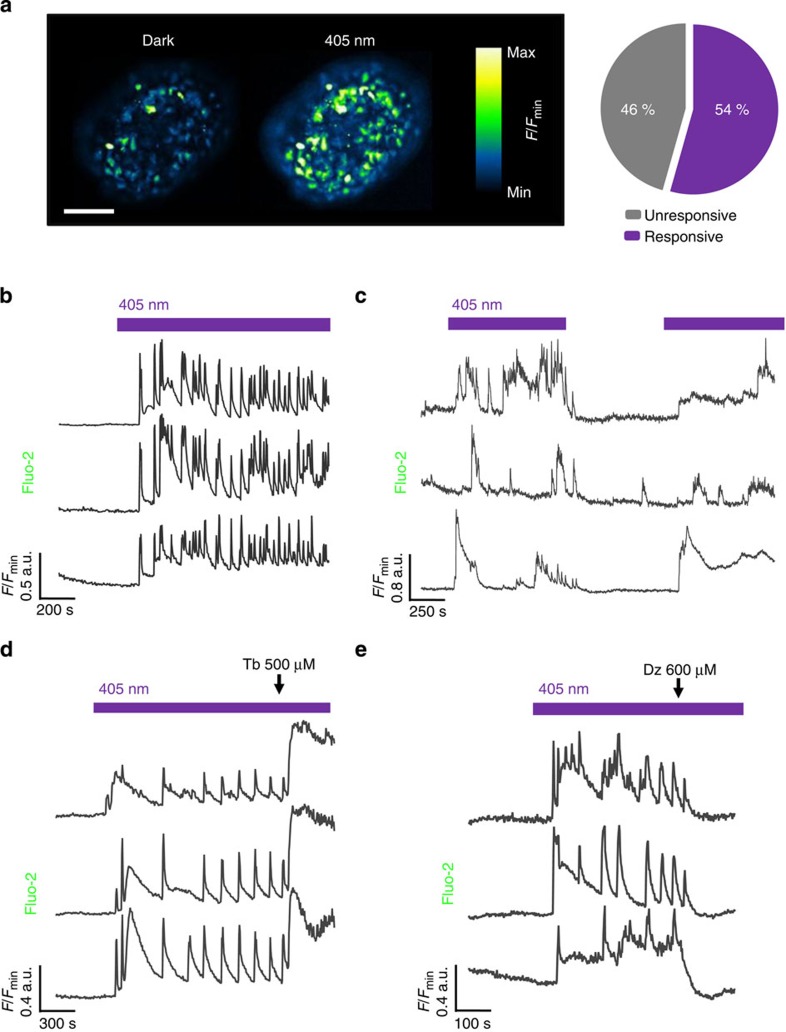
JB253 allows optical manipulation of pancreatic beta cell activity. (**a**) Beta cells residing within JB253-treated islets display large increases in cytosolic Ca^2+^ following exposure to 405 nm (purple) (scale bar, 110 μm) (linear look up table (LUT)). (**b**) Illumination with 405 nm induces large and synchronous rises in Ca^2+^ as indicated by Fluo-2 (representative trace from *n*=10 recordings). (**c**) JB253 can be used to impose complex dynamics including Ca^2+^ oscillations (representative trace from *n*=5 recordings). (**d**) High-dose tolbutamide (Tb) augments the effects of JB253 on K_ATP_ channel blockade (representative traces from *n*=6 recordings). (**e**) Diazoxide (Dz) inhibits JB253 effects by opening the K_ATP_ channel ion pore (representative traces from *n*=4 recordings).

Using functional multicellular Ca^2+^ imaging to monitor cell activity directly *in situ* within intact islets^30^,^31^, increases in cytosolic free Ca^2+^, assumed largely to emanate from beta cells under the conditions used here^32^, could be evoked following global illumination using a 405-nm laser (Fig. 4a) (*n*=10 recordings). Just over half (54%) of the Fluo-2-loaded population responded to illumination with synchronous Ca^2+^ rises (Fig. 4a,b). Demonstrating the utility of JB253 for the fine control of beta cell function, discrete Ca^2+^ oscillations, thought to underlie generation of insulin pulses^33^,^34^, could be imposed using repeat exposure to 405 nm (Fig. 4c). As anticipated, high doses of tolbutamide and diazoxide were able to augment and suppress, respectively, the effects of JB253 (Fig. 4d,e) (*n*=4–6 recordings).

**Figure 5:**
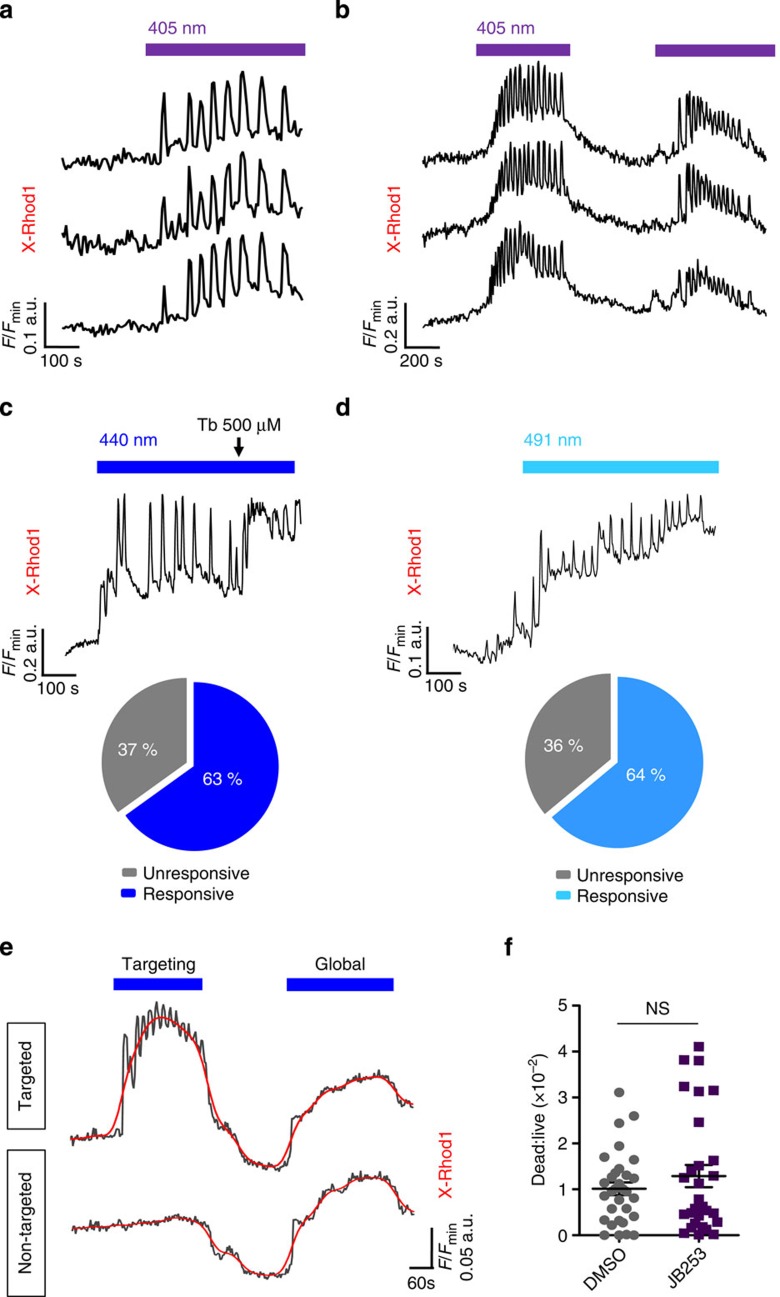
Specific photoswitching of beta cell function in the violet-blue spectrum. (**a**) JB253-treated cells loaded with the red-shifted Ca^2+^ indicator X-Rhod1 (*λ* excitation=561 nm) similarly respond to 405 nm with Ca^2+^ rises (representative traces from *n*=3 recordings). (**b**) As in **a**, but imposition of oscillations (representative traces from *n*=3 recordings). (**c**) JB253-treated beta cells display large increases in cytosolic Ca^2+^ following exposure to 440 nm (representative trace from *n*=6 recordings). (**d**) As in **c**, but following illumination with 491 nm (Tb, tolbutamide; positive control) (representative trace from *n*=5 recordings). (**e**) A single islet can be photoswitched using a targeting laser while leaving its neighbour quiescent (~200 μm center–center) (representative traces from *n*=3 recordings). A global laser pulse evokes activity in both islets (grey, raw; red, smoothed). (**f**) Incubation of islets with JB253 does not alter cell viability as assessed by calcein-AM and propidium iodide incorporation (NS, not significant versus DMSO alone, Student’s *t*-test) (*n*=28 islets from four animals). Values represent mean±s.e.m.

The wavelength required to excite Fluo-2 (*λ*=491 nm), a commonly used Ca^2+^ indicator, could potentially lead to K_ATP_ channel closure in JB253-treated islets due to *cis*-isomer formation. We therefore decided to repeat the above experiments using the red (*λ*=561 nm)-excited Ca^2+^ indicator X-Rhod1. Identical results were obtained for studies with Fluo-2 (Fig. 5a,b) (63% responsive X-Rhod1-loaded cells) (*n*=3 recordings), confirming that the photostationary state of JB253 during brief (263 ms) pulses of 491-nm light was alone insufficient to close K_ATP_ channels in the islet preparation. Likewise, global Ca^2+^ oscillations could be induced in JB253-treated islets by more prolonged illumination with 440-nm and 491-nm laser lines and, as expected from the electrophysiological recordings, both wavelengths appeared to activate a slightly larger cell population (Fig. 5c,d). These observations were unlikely due to K_ATP_ channel closure at 561 nm, since JB253 was unable to photoswitch K^+^ currents at wavelengths >560 nm (Supplementary Fig. 8).

Demonstrating the spatial precision of JB253, a single islet from a doublet could be activated using a targeting laser without significantly stimulating its neighbour (~200 μm from center to center) (Fig. 5e), in part aided by the high molecular extinction coefficient (38,670 mol^−1^ cm^−1^ at 485 nm; see Supplementary Fig. 9). Lastly, JB253 did not appear to be cytotoxic to islets, as necrosis indices showed no significant differences in cell death versus DMSO alone (Fig. 5f).

### Manipulation of human islet function using JB253

**Figure 6:**
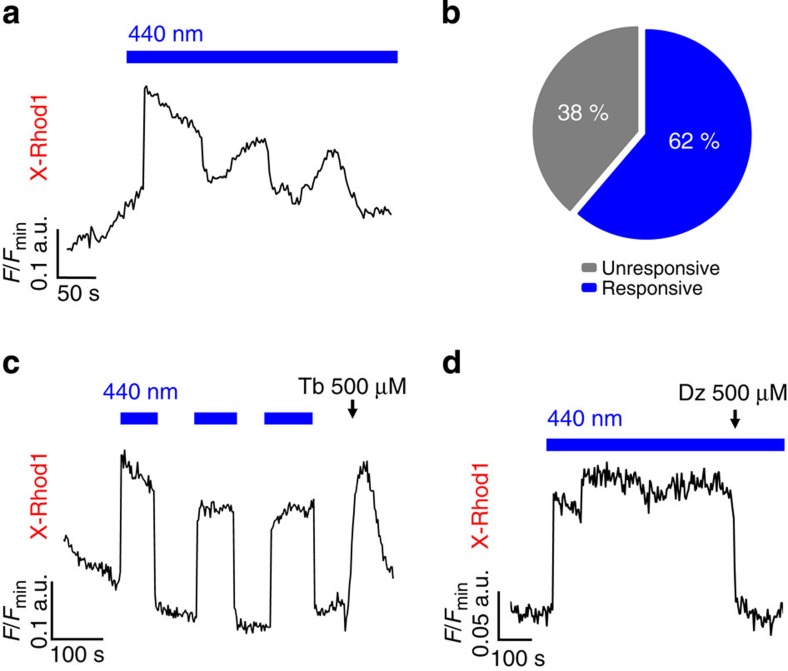
Manipulation of human islet activity using JB253. (**a**) JB253-treated beta cells residing within intact human islets respond to 440 nm with rapid rises in cytosolic Ca^2+^ levels (representative trace from *n*=8 recordings). (**b**) Almost 62% of X-Rhod1-loaded cells respond to JB253 with Ca^2+^ rises. (**c**) As in **a**, but imposition of oscillations to demonstrate reversibility of JB253 effects in human tissue (Tb, tolbutamide; positive control) (representative trace from *n*=4 recordings). (**d**) Reversal of JB253 action using diazoxide to open the K_ATP_ channel ion pore (Dz, diazoxide; negative control) (representative trace from *n*=5 recordings). In all cases, islets were derived from three donors.

To underline the translational potential of JB253 for use in man, Ca^2+^-imaging experiments were repeated using isolated human islets of Langerhans. As observed for their mouse counterparts, beta cells within JB253-treated human islets responded to 440 and 491 nm with large intracellular Ca^2+^ rises, and oscillations could be coaxed simply by turning the laser on and off (Fig. 6a–c). JB253 effects appeared to be due to K_ATP_ blockade, as they could be mimicked and reversed using tolbutamide and diazoxide, respectively (Fig. 6c,d).

### Optical stimulation of insulin release using JB253

**Figure 7:**
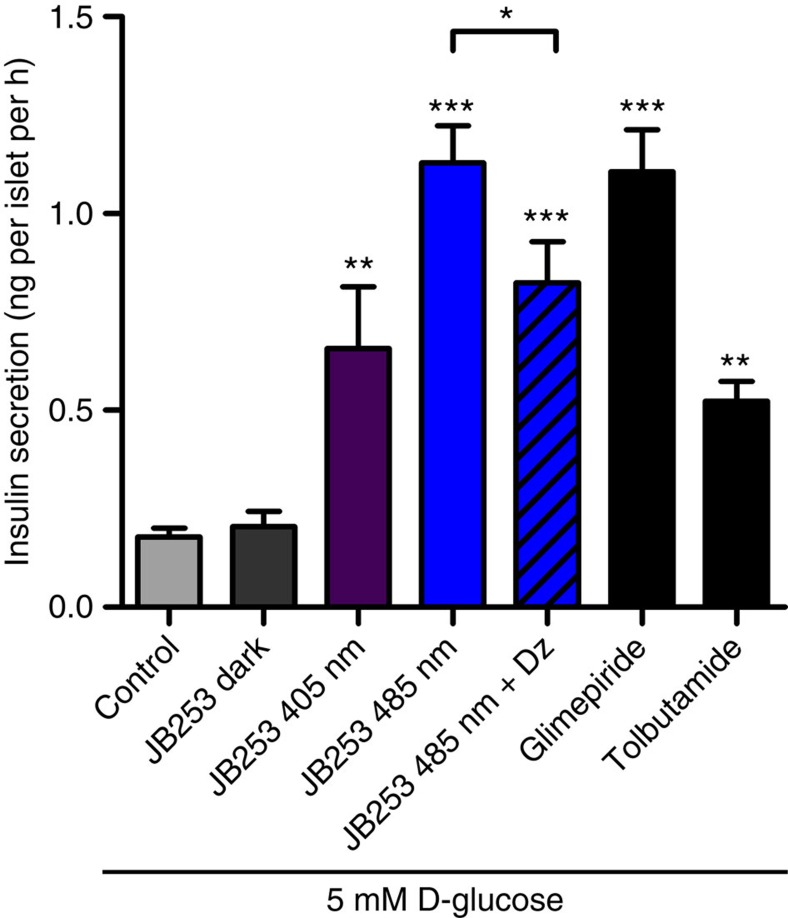
JB253 yields optical control of insulin secretion. Application of JB253 under dark conditions is unable to influence insulin release versus control (5 mM glucose alone; G5) during static incubation of isolated murine islets. By contrast, illumination with 405 and 485 nm significantly increases insulin secretion, and this is similar in magnitude to that achieved using glimepiride and tolbutamide (*n*=9 mice) (*P<0.05 versus JB253 485 nm; **P<0.01 and ***P<0.001 versus G5; one-way analysis of variance). Values represent mean±s.e.m.

To cement the link between photocontrol of K_ATP_ channels, [Ca^2+^]_i_ and insulin secretion, islets (from *n*=9 mice) were incubated in the presence of JB253 while exposing to either dark (no illumination), 405 nm or 485 nm. Insulin release was similar in control experiments (5 mM glucose, shown to sensitize beta cells to sulfonylurea^35^) and JB253-treated islets in the dark, suggesting that any K_ATP_ channel block and VDCC activity detected under these conditions was subthreshold for triggering Ca^2+^-activated exocytosis (Fig. 7). By contrast, JB253-treated islets secreted almost four- to eightfold more insulin following illumination, and this could be partially reversed using diazoxide (Fig. 7). When exposed to 485 nm light, JB253 was equipotent to glimepiride at stimulating insulin secretion (Fig. 7).

## Discussion

In the present manuscript, we have described the development and testing of JB253, a chemical chimera of glimepiride and an azobenzene, which allows light-induced closure of K_ATP_ channels. In the primary tissue employed here, viz islets of Langerhans, this translates to activated Ca^2+^ flux and insulin release.

The principles of photopharmacology, that is, the control of biological function with small-molecule photoswitches, are now well established^25^,^36^,^37^. In particular, azobenzene photoswitches have been employed as photochromic neurotransmitters and neuromodulators^38^,^39^, ion channel blockers^24^,^40^, covalently bound ion channel gates^41^ and enzyme inhibitors^42^,^43^. With respect to ion channels, however, they have mostly been used to optically control excitable cells in the mammalian nervous system, and none have directly targeted K_ATP_ channels. These are ubiquitously expressed channels that contribute to membrane potential in a number of cell types including hypothalamic and hippocampal neurons, cardiac myocytes, vascular smooth muscle and neuroendocrine cells^11^,^20^,^21^,^22^,^23^,^44^. Importantly, K_ATP_ channels translate metabolic state to transmembrane potential and, in pancreatic beta cells, are central to glucose-stimulated insulin secretion^10^,^11^,^12^. Since electrical status is generally correlated to biological output in excitable tissues, JB253 may provide a useful tool for investigating K_ATP_ channel function under a range of normal and pathological states.

The prevailing view of sulfonylurea action is one of SUR1 binding, K_ATP_ channel closure and alterations to beta cell membrane potential^7^,^8^,^9^. However, recent studies have also invoked a K_ATP_ channel-independent signalling pathway whereby sulfonylurea may alter insulin release via Epac2A interactions^27^,^28^,^29^. Using radioactive displacement assays in combination with FRET experiments, JB253 was found to interact with both SUR1 and Epac2A. While SUR1 affinity for JB253 was much lower than glimepiride, we were unable to properly assess the active *cis*-state due to rapid thermal back-relaxation. As such, a role for illumination in strengthening any interaction cannot be excluded, for example, by altering binding conformation due to isomerization. Nonetheless, JB253 and glimepiride possess similar EC_50_ values for intracellular Ca^2+^ rises and, when applied at the same concentration, both compounds stimulated almost identical levels of insulin secretion. Thus, JB253 possesses a similar activity profile to glimepiride, most likely due to signalling via pathways generally acknowledged to underlie sulfonylurea action.

In addition to photopharmacology, optogenetic and artificial light-sensitive K^+^ channels are equally applicable to the remote control of electrically responsive cells, including beta cells^45^,^46^. However, therapeutic potential in humans is limited by the requirement for genetic manipulation, high activation irradiances and the hyperpolarizing effects of recombinantly expressed Kir6.2. Alternatively, an implantable synthetic optogenetic transcription device has recently been shown to improve blood-glucose homeostasis in a mouse model of T2DM via the expression and secretion of incretin^47^. However, debate still exists as to whether incretin-based therapies are associated with increased risk of pancreatitis and pancreatic adenocarcinoma^48^,^49^. Nonetheless, similar concepts have recently been extended to designer fusion molecules and may in the future be adopted for insulin release^50^. By contrast, due to its favourable profile as an exogenously applied sulfonylurea that is sensitive to light, JB253 has advantages both as a research tool and as an anti-diabetic agent. We note, however, that confirmation of glucose-lowering effects in rodents is required before studies using JB253 can be extended to man.

In the context of photodynamic therapy, light penetration in human tissues has been studied in detail and is now well understood^51^. Although, the current activation wavelength of JB253 (400–500 nm) limits deep tissue penetration, for example, through the skin, we have recently begun to develop variants that can be switched at longer wavelengths. In addition, stimulated by the brisk development of optogenetics^52^, devices that can deliver light to target tissues with minimal invasiveness and high spatial precision have emerged^53^,^54^, although their application to the pancreas is untested.

We therefore speculate that JB253, or related photoswitchable molecules, which regulate K_ATP_ channels, may have an impact on human medicine and research. A long-standing challenge in endocrinology has been the inability to properly recreate the dynamics that underlie pulsatile hormone release, a prerequisite for proper downstream organ function^55^. Furthermore, disparate biological systems can use similar or identical molecular components. For example, K_ATP_ channels are also expressed in the heart and brain, including in neuronal populations tasked with the central regulation of glucose homeostasis and counterregulatory responses^20^,^56^. Photopharmacology has the ability to target drug activity to the primary site of dysfunction with high spatial and temporal resolution^37^. JB253 holds particular promise in this regard. It is non-cytotoxic and can be used to repeatedly modulate rodent and human beta cell activity, the basis for recreating the oscillatory activity known to orchestrate hormone pulses^33^,^34^. Its light dependency means that JB253 activity is spatially restricted by illumination, potentially reducing extra-pancreatic effects. Finally, the ability to ‘turn on’ or ‘turn off’ JB253 action would allow insulin secretion to be tailored to peak demand. Therefore, pending thorough *in vivo* validation, JB253 and its congeners could potentially open up new avenues for the treatment of T2DM.

In summary, we have designed and synthesized a light-sensitive sulfonylurea, JB253, which has a broad spectrum of application due to conferment of photoswitching on K_ATP_ activity.

## Methods

### Chemical synthesis

*(*E*)-4-((4-(Diethylamino)phenyl)diazenyl)benzenesulfonamide* (1). Sulfanilamide (2.00 g, 11.61 mmol, 1.0 eq.) was dissolved in 2.4 M HCl and cooled to 0 °C. Under vigorous stirring, a solution of NaNO_2_ (0.96 g, 13.91 mmol, 1.2 eq.) in 6 ml water was added dropwise until the solution turned pale yellow. The formed diazonium salt was stirred under ice-cooling for an additional 10 min before it was transferred into a solution of *N*,*N*-diethylaniline (1.73 g, 11.61 mmol, 1.84 ml, 1.0 eq.) in a 1/1 mixture of 1 M NaOAc/MeOH. The solution turned to dark red and was allowed to warm to room temperature under stirring. The crude product was extracted with EtOAc (3x), and the combined organic layers were washed with brine and dried over MgSO_4_. Flash column chromatography (25% EtOAc/*i*-hexanes) yielded 1.45 g (4.37 mmol) of the desired product as a red powder in 38% yield. ^**1**^H NMR (400 MHz, DMSO-d_6_): *δ* (p.p.m.)=7.95 (d, *J*=8.6 Hz, 2H), 7.88 (d, *J*=8.6 Hz, 2H), 7.81 (d, *J*=9.2 Hz, 2H), 7.45 (s, 2H), 6.82 (d, *J*=9.3 Hz, 2H), 3.47 (q, *J*=7.0 Hz, 4H), 1.15 (t, *J*=7.0 Hz, 6H). ^13^C NMR (101 MHz, DMSO-d_6_): *δ* (p.p.m.)=154.2, 150.8, 143.8, 142.2, 126.9, 125.8, 121.9, 111.1, 44.2, 12.5. High-resolution mass spectrometry (electrospray ionization): calc. for C_16_H_21_N_4_O_2_S^+^ (M+H)^+^: 333.1380, found: 333.1377. *R*_t_ (liquid chromatography–mass spectrometry (LC-MS); MeCN/H_2_O/formic acid=10/90/0.1→90/10/0.1 over 7 min)=4.364 min. Ultraviolet**/**Vis (LC-MS): *λ*_max_=460 nm.

*(E)-N-(Cyclohexylcarbamoyl)-4-((4-(diethylamino)phenyl)diazenyl)benzenesulfonamide (JB253)*. A mixture of (*E*)-4-((4-(diethylamino)phenyl)diazenyl)benzenesulfonamide (332 mg, 1.0 mmol, 1.0 eq.) and Cs_2_CO_3_ (1.30 g, 4.0 mmol, 4.0 eq.) in acetone (20 ml) was refluxed for 1 h before addition of cyclohexyl isocyanate (125 mg, 1.0 mmol, 119 μl, 1.0 eq.) diluted in acetone (20 ml). The reaction mixture was refluxed for an additional 3 h, before cooling to ~40 °C. The crude solid was filtered and washed with small amounts of acetone before it was carefully dissolved in MeOH to yield 450 mg (0.98 mmol) of JB253 product in 98% yield. ^1^H NMR (400 MHz, DMSO-d_6_): *δ* (p.p.m.)=7.83 (d, *J*=8.5 Hz, 2H), 7.78 (d, *J*=9.1 Hz, 2H), 7.69 (d, *J*=8.5 Hz, 2H), 6.80 (d, *J*=9.3 Hz, 2H), 5.62 (br s, 2H), 3.46 (q, *J*=7.0 Hz, 4H), 3.20 (br s, 1H), 1.86–1.38 (m, 5H), 1.33–0.92 (m, 11H). ^13^C NMR (101 MHz, DMSO-d_6_): *δ* (p.p.m.)=172.7 (heteronuclear multiple-bond correlation (HMBC), see Supplementary Fig. 4), 152.6, 150.2, 148.5, 142.2, 127.4, 125.3, 120.8, 111.0, 47.8, 44.1, 33.5, 25.5, 24.9, 12.5. High-resolution mass spectrometry (electrospray ionization): calc. for C_23_H_32_N_5_O_3_S^+^ (M+H)^+^: 458.2220, found: 458.2219. *R*_t_ (LC-MS; MeCN/H_2_O/formic acid=10/90/0.1→90/10/0.1 over 7 min)=5.285 min. Ultraviolet**/**Vis (100 μM in DMSO): *λ*_max_=472 nm; (LC-MS): *λ*_max_=468 nm. *ε*_405_ _nm_=18,501 mol^−1^ cm^−1^; *ε*_485_ _nm_=38,670 mol^−1^ cm^−1^. Infrared (attenuated total reflectance): wavenumber in cm^−1^=3331, 2928, 2851, 1652, 1626, 1602, 1576, 1537, 1514, 1390, 1349, 1174, 1130, 1086, 1042, 843, 820, 676. m.p.=190 °C.

### General chemistry

Flash column chromatography was carried out on silica gel 60 (0.040–0.063 mm) purchased from Merck. Reverse phase flash column chromatography was carried out on Waters C18 silica gel (0.055–0105, mm, 125 Å). Reactions and chromatography fractions were monitored by thin-layer chromatography on Merck silica gel 60 F254 glass plates. The spots were visualized either under ultraviolet light at 254 nm or with appropriate staining method (iodine, *p*-anisaldehyde, KMnO_4_) followed by heating.

NMR spectra were recorded in deuterated solvents on Varian Mercury 200, Bruker AXR 300, Varian VXR 400S, Bruker AMX 600 and Bruker Avance III HD 400 (equipped with a CryoProbe) instruments and calibrated to residual solvent peaks (^1^H/^13^C in p.p.m.): DMSO-d_6_ (2.50/39.52). Multiplicities are abbreviated as follows: s=singlet, d=doublet, t=triplet, q=quartet, br=broad and m=multiplet. Spectra are reported based on appearance, not on theoretical multiplicities derived from structural information.

A Varian MAT CH7A mass spectrometer was used to obtain low- and high-resolution electron impact mass spectra. Low- and high-resolution ESI mass spectra were obtained on a Varian MAT 711 MS instrument operating in either positive or negative ionization modes.

Solvents for column chromatography and reactions were purchased in HPLC grade or distilled over an appropriate drying reagent before use. If necessary, solvents were degassed either by freeze-pump-thaw or by bubbling N_2_ through the vigorously stirred solution for several minutes. Unless otherwise stated, all other reagents were used without further purification from commercial sources.

Ultraviolet/Vis spectra were recorded on a Varian Cary 50 Bio UV-Visible Spectrophotometer using Helma Suprasil precision cuvettes (10 mm light path).

LC-MS was performed on an Agilent 1260 Infinity HPLC System, MS-Agilent 1100 Series, Type: 1946D, Model: SL, equipped with a Agilent Zorbax Eclipse Plus C18 (100 × 4.6 mm, particle size 3.5 micron) RP column.

Infrared spectra were recorded on a PerkinElmer Spectrum BX-59343 instrument as neat materials. For detection, a Smiths Detection DuraSam-plIR II Diamond ATR sensor was used. The measured wavenumbers are reported in cm^−1^.

Melting points were measured on an EZ-Melt apparatus (Stanford Research Systems) and are uncorrected.

Extinction coefficients were measured on a BMG Labtech Omega Series FLUOstar microplate reader with clear flat-bottom white 96-well plates by full spectra acquirement in low-K^+^ external bath buffer (containing in mM: 3 KCl, 118 NaCl, 25 NaHCO_3_, 2 CaCl_2_, 1 MgCl_2_, 10 HEPES; NaOH to pH 7.4). All JB253**-**containing solutions (dilution series, *n*=4: 10 nM; 100 nM; 1 μM; 10 μM; 25 μM; 50 μM) were background substracted and fitted with a linear slope. Volumes were 100 μl each, which resulted in a path length of *l*=2.94 mm. *pK*_*a*_ measurements and data processing were performed using the same instrument and protocol as in the study by Martinez and Dardonville^57^. Full absorbance spectra (280–800 nm) were acquired and background subtracted before spectral differences were calculated. The total change of maximal positive and maximal negative difference was calculated and plotted against pH. Sigmoidal fit of the obtained plot gave access to the *pK*_*a*_.

### Crystallography

X-Ray data collection was performed on a Bruker D8Venture at 173 K using MoKα-radiation (*λ*=0.71073 Å). The APEX2 (v2012.4-3, Bruker AXS Inc.) software and embedded programs were applied for the integration, scaling and multi-scan absorption correction of the data. The structures were solved by direct methods with SIR97^58^ and refined by least-squares methods against F2 with SHELXL-97^59^. All non-hydrogen atoms were refined anisotropically. The C-bound hydrogen atoms were placed in ideal geometry riding on their parent atoms, *N*-bound hydrogen atoms were refined freely. The crystallographic data for JB253 is available free of charge from The Cambridge Crystallographic Data Centre via www.ccdc.cam.ac.uk/data_request/cif (accession ref. CCDC 1014606).

### Electrophysiology

HEK293t cells (obtained from the Leibniz-Institut DSMZ: #305) were incubated in Dulbecco’s modified Eagle’s medium+10% foetal bovine serum and used for electrophysiological recordings 24–48 h following Lipofectamine transfection with plasmids encoding Kir6.2 (Genbank D50581), rat SUR1 (Genbank L40624) and green fluorescent protein. Whole-cell patch-clamp experiments were performed using a standard electrophysiology setup equipped with a HEKA Patch Clamp EPC10 USB amplifier and PatchMaster software (HEKA Electronik). Micropipettes were generated from ‘Science Products GB200-F-8P with filament’ pipettes using a vertical puller. Resistance varied between 5 and 7 MΩ. Bath solution contained in mM: 3 KCl, 118 NaCl, 25 NaHCO_3_, 2 CaCl_2_, 1 MgCl_2_, 10 HEPES (NaOH to pH 7.4). Pipette solution contained in mM: 90 K-gluconate, 10 NaCl, 10 KCl, 1 MgCl_2_, 10 EGTA, 60 HEPES (KOH to pH 7.3), and the holding potential was −60 mV. Illumination during electrophysiology and ultraviolet/Vis experiments was provided by a TILL Photonics Polychrome 5000 monochromator. JB253 was applied at 50 μM, as this concentration was found to be maximal for stimulating Ca^2+^ rises.

MIN6 cells (a kind gift from Dr Jun-ichi Miyazaki, Osaka University) were cultured in Dulbecco’s modified Eagle’s medium supplemented with 15% foetal bovine serum, 1% glutamine, 2% HEPES buffer, 0.0005% β-mercaptoethanol and 1% penicillin/streptomycin. Cells were seeded onto glass coverslips 24 h before whole-cell patch-clamp experiments using micropipettes generated from thin-walled borosilicate capillaries (3–6 MΩ). Bath solution contained in mM: 3 KCl, 118 NaCl, 25 HEPES, 3 MgCl_2_ and 2 CaCl_2_. Pipette solution contained in mM: 150 KCl, 3 MgCl_2_, 5 EGTA, 10 HEPES, 0.3 K_2_ATP (pH 7.2), and the holding potential was −60 mV. Illumination was provided using a X-Cite 120 mercury arc lamp (Lumen Dynamics) with a bandpass filter (470±20 nm). Voltage ramps from −20 mV to −120 mV (500 ms duration) were applied every 5 s to produce current–voltage relationships in the presence and absence of JB253. All cells lines were regularly mycoplasma tested.

### Mouse islet isolation

Male and female CD1 and C57BL6 mice (8–20 weeks) were maintained in a specific pathogen-free facility under a 12 h light–dark cycle with *ad libitum* access to water and food. Animals were euthanized using a schedule-1 method and pancreatic islets isolated by collagenase digestion. All procedures were regulated by the Home Office according to the Animals (Scientific Procedures) Act 1986 of the United Kingdom (PPL 70/7349), and study approval granted by the Animal Welfare and Ethical Review Body of Imperial College. No randomization was used for animal experimentation, since mice were only used as tissue donors.

### Human islet isolation

Human islets were isolated from deceased heart-beating donors (*n*=3) at transplantation facilities in Pisa and Edmonton with the relevant national and local ethical permissions, including consent from next of kin where required, and cultured in Roswell Park Memorial Institute medium supplemented with 5.5 mM D-glucose, 10% foetal calf serum, 100 U/ml penicillin, 100 μg/ml streptomycin and 0.25 μg/ml fungizone (37 °C, 5% CO_2_). All studies involving human tissue were approved by the National Research Ethics Committee London (Fulham), REC #07/H0711/114.

### Calcium imaging

Islets were loaded for 30–45 min in Fluo2-AM (10 μM) or for 2–5 min with X-Rhod1 (5 μM) diluted with a mixture of DMSO (0.01%, wt/vol) and pluronic acid (0.001%, wt/vol; all Invitrogen) in a bicarbonate buffer containing in mM: 120 NaCl, 4.8 KCl, 1.25 NaH_2_PO_4_, 24 NaHCO_3_, 2.5 CaCl_2_, 1.2 MgCl_2_ and 5 D-glucose. Functional multicellular Ca^2+^ imaging was performed using a Zeiss Axiovert M200 fitted with a Nipkow spinning-disk head (Yokogawa CSU-10) and a × 10/0.3 numerical apperture objective adjusted for chromatic aberration (EC Plan-Neofluar, Zeiss). Pulsed excitation (frequency=0.5 Hz; exposure=263 ms) was delivered at 491 nm and emitted signals recorded at 500–550 nm with a back-illuminated 16-bit EM-CCD camera (ImageEM 9100-13; Hamamatsu). During recording, islets were maintained at 35–36 °C in the presence of 50 μM JB253 using a custom-manufactured perfusion and heating system (Digital Pixel). Drugs were introduced through the perfusion system at the indicated time points and concentrations. Violet light was delivered by a 405±5-nm laser coupled to the side port of the microscope and configured to fill the back of the objective with light using an Optospot (Cairn Research). Blue light was delivered using 440±5-nm and 491±5-nm diode lasers controlled by a laser merge module (Spectral Applied Research) to allow simultaneous exposure and acquisition. For single islet targeting, a 473±5 nm laser was coupled to a custom-manufactured dichroic array (Cairn Research), allowing user-directed steering of a collimated laser spot across the field of view. Signals were normalized using *F*/*F*_min_ where *F* is fluorescence at a given time point and *F*_min_ is minimum fluorescence.

### Cytotoxicity assay

Islets were incubated with either DMSO or JB253 for 1 h before staining with 3 μM of calcein-AM (live) and 2.5 μM of propidium iodide (dead). Absorbance/emission was detected at 491/525 and 561/620 nm for calcein and PI, respectively. The area of dead:live cells was calculated as a unitary ratio and the observer blinded to treatment identity.

### Epac2 imaging

For Epac2 imaging, HEK293t were transfected with the full-length construct for Epac2-camps containing the cAMP- and sulfonylurea-binding domains^29^ (a kind gift from Prof. Jin Zhang, Johns Hopkins University) before imaging^60^ using a HEPES-bicarbonate buffer containing in mM: 120 NaCl, 4.8 KCl, 24 NaHCO_3_, 0.5 Na_2_HPO_4_, 5 HEPES, 2.5 CaCl_2_, 1.2 MgCl_2_ and 5 D-glucose. Excitation was delivered at 440 nm and emitted signals captured using cerulean (530 nm) and citrine (470 nm) filters. FRET was calculated as the ratio of Cerulean (CFP):Venus (YFP) fluorescence. Signals were normalized using *R*/*R*_o,_ where *R* is the ratio at a given time point and *R*_o_ is the minimum ratio.

### Measurements of insulin secretion from isolated islets

Insulin secretion was measured from six islets per well, incubated at 37 °C for 30 min in 0.5 ml of Krebs-HEPES-bicarbonate solution (containing in mM: 130 NaCl, 3.6 KCl, 1.5 CaCl_2_, 0.5 MgSO_4_, 0.5 NaH_2_PO_4_, 2 NaHCO_3_, 10 HEPES and 0.1% (wt/vol) bovine serum albumin, pH 7.4) containing the indicated glucose concentration and tolbutamide (100 μM), glimepiride (50 μM), diazoxide (250 μM) and JB253 (50 μM). Illumination (*λ*=405±20 nm and 485±6 nm) was performed using a Fluostar Optima microplate reader (BMG Labtech) set to deliver 30 s of light every 2 min to the designated wells. Insulin concentrations were determined in duplicate using specific radioimmunoassay (EMD Millipore).

### [3H]-Glibenclamide radioassay

SUR1-expressing HEK293t cells were harvested and washed twice in assaying buffer containing in mM: 119 NaCl, 4.7 KCl, mM CaCl_2_, 1.2 KH_2_PO_4_, 1.2 MgSO_4_, 5 NaHCO_3_ and 20 HEPES, pH 7.4. In a 96-well plate, ~200,000 cells per well were incubated for 50 min with [3H]-glibenclamide (PerkinElmer) and different concentrations of glimepiride (Sigma-Aldrich) or JB253. Incubation was terminated by rapid filtration through Whatman GF/C filters by means of a Brandel MWXR-96 TI harvester and filters were washed three times with ice-cold assay buffer. Radioactivity was counted 6 h after cell and filter lysis in 200 μl Rotiszint EcoPlus (Roth) using a Packard microbeta scintillation counter (PerkinElmer).

### Statistical analysis

Data distribution was determined using the D’Agostino omnibus test. Non-multifactorial pairwise comparisons were made using the Student’s *t*-test. Interactions between multiple treatments were assessed using one-way analysis of variance followed by pairwise comparisons using Bonferroni’s *post hoc* test. Nonlinear regression was used to calculate the EC_50_ of normalized and log-transformed concentration–response curves. For [3H]-glibenclamide displacement assays, data points were fitted to the Hill equation before calculation of the halfmax value. In all cases, analysis was performed using Graphpad Prism (Graphpad Software) and IgorPro, and experimental numbers reported as independent biological replicates. No animals or data were excluded from the analysis and results were considered significant at *P*<0.05. Effect sizes in islets/cells are usually sufficiently large that multiple animals/independent replication is a more important determinant of power in studies requiring statistical comparison.

## Additional information

**How to cite this article**: Broichhagen, J. *et al*. Optical control of insulin release using a photoswitchable sulfonylurea. *Nat. Commun.* 5:5116 doi: 10.1038/ncomms6116 (2014).

**Accession codes**: Crystallographic data for JB253 have been deposited in The Cambridge Crystallographic Data Centre under accession code CCDC 1014606.

## Supplementary Material

Supplementary Information Supplementary Figures 1-9 and Supplementary Tables 1-4
