# Supplementary material for: Optical control of insulin release using a photoswitchable sulfonylurea
Source: Nat Commun. 2014 Oct 14;5:5116. doi: 10.1038/ncomms6116 (PMC4208094; doi:10.1038/ncomms6116)
Supplement: Supplementary Information — Supplementary Figures 1-9 and Supplementary Tables 1-4 [file ncomms6116-s1.pdf]

SUPPLEMENTARY FIGURES

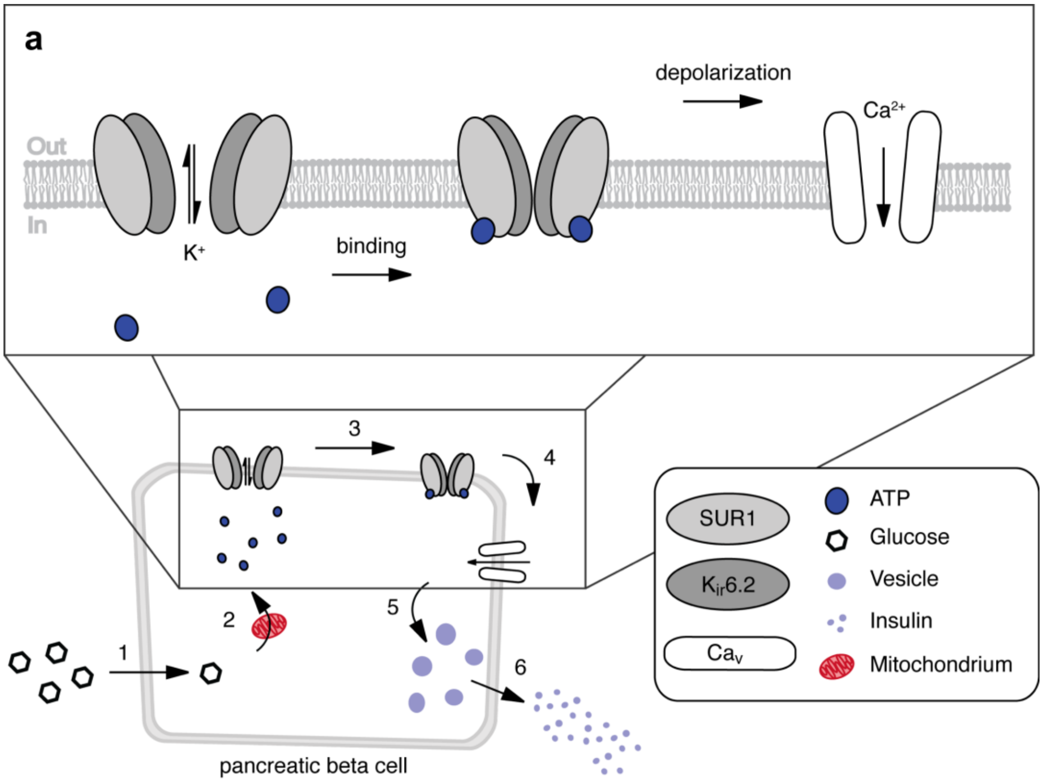

Supplementary Figure 1: Stimulation-secretion coupling in the pancreatic beta-cell. Glucose is transported into the cell and undergoes glycolysis followed by mitochondrial metabolism (**1**). The increase in ATP:ADP ratio (**2**) blocks hyperpolarizing K_{ATP} channels. The resulting depolarization (**3**) of the cell membrane leads to Ca^{2+} influx (**4**), triggering exocytosis (**5**) and release of insulin into the circulation (**6**). **a)** Zoom-in of K_{ATP} channel function.

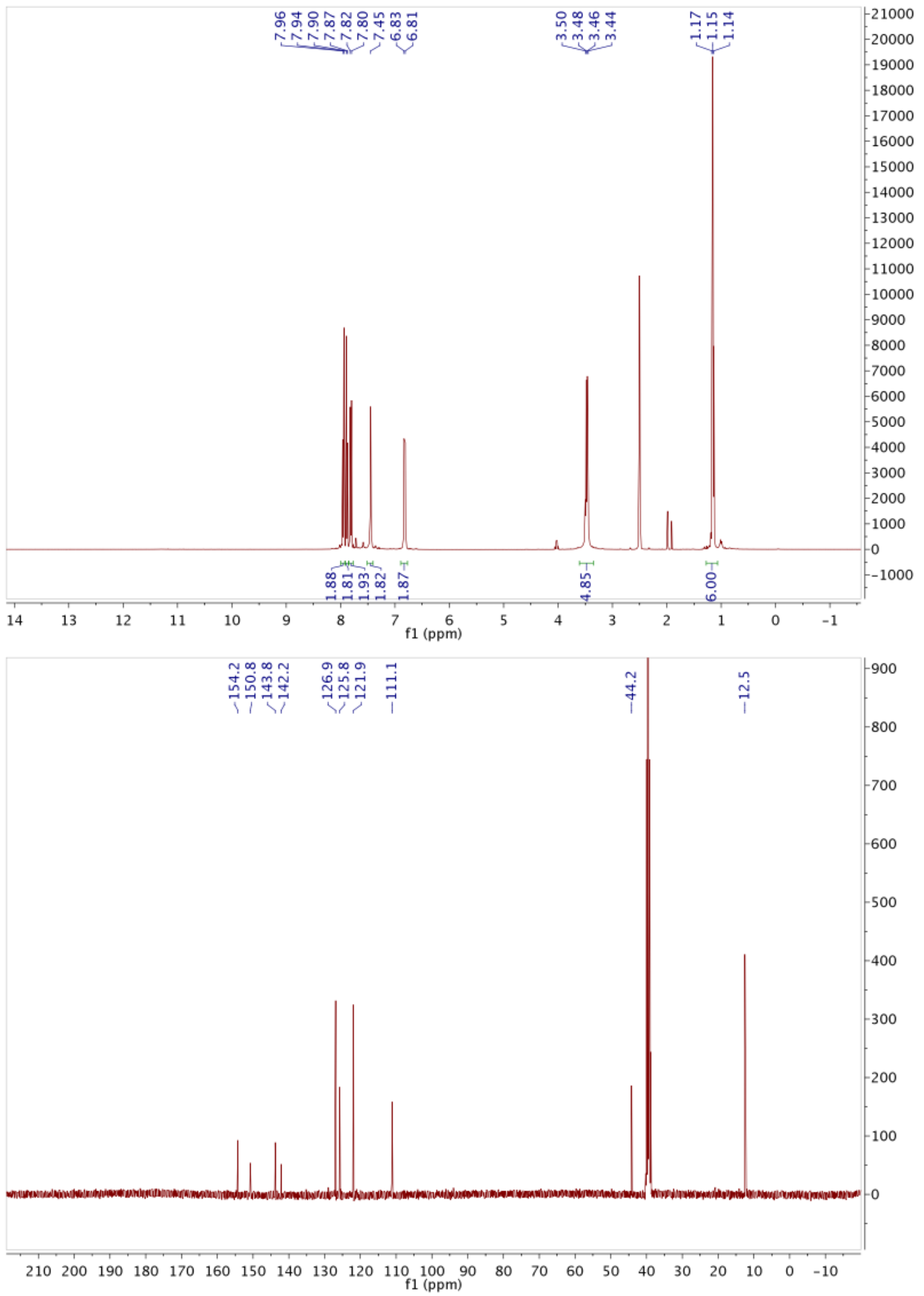

Supplementary Figure 2: ^{1}H and ^{13}C NMR of (*E*)-4-((4-(diethylamino)phenyl)diazenyl)benzenesulfonamide.

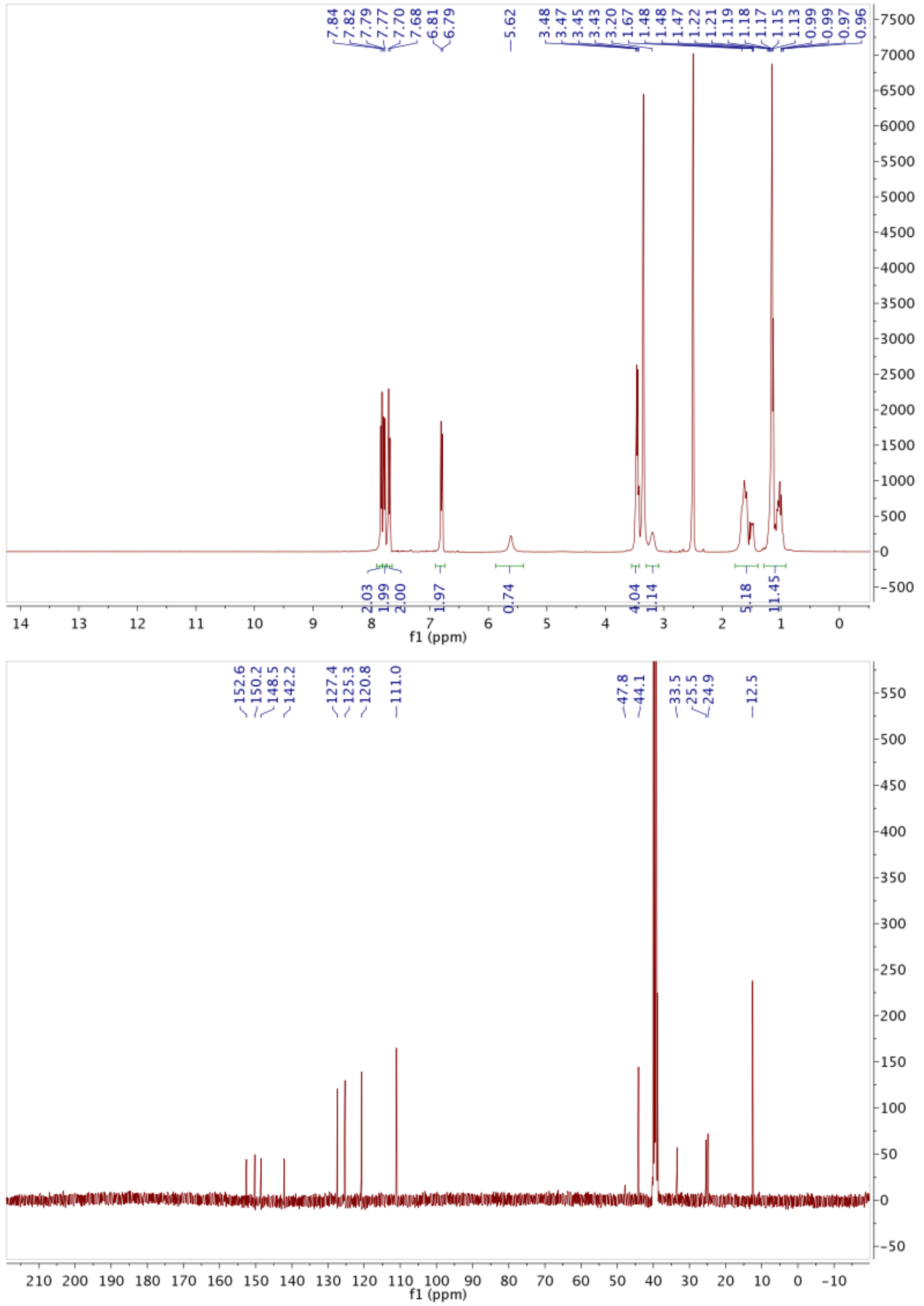

Supplementary Figure 3: ^{1}H and ^{13}C NMR of **JB253**.

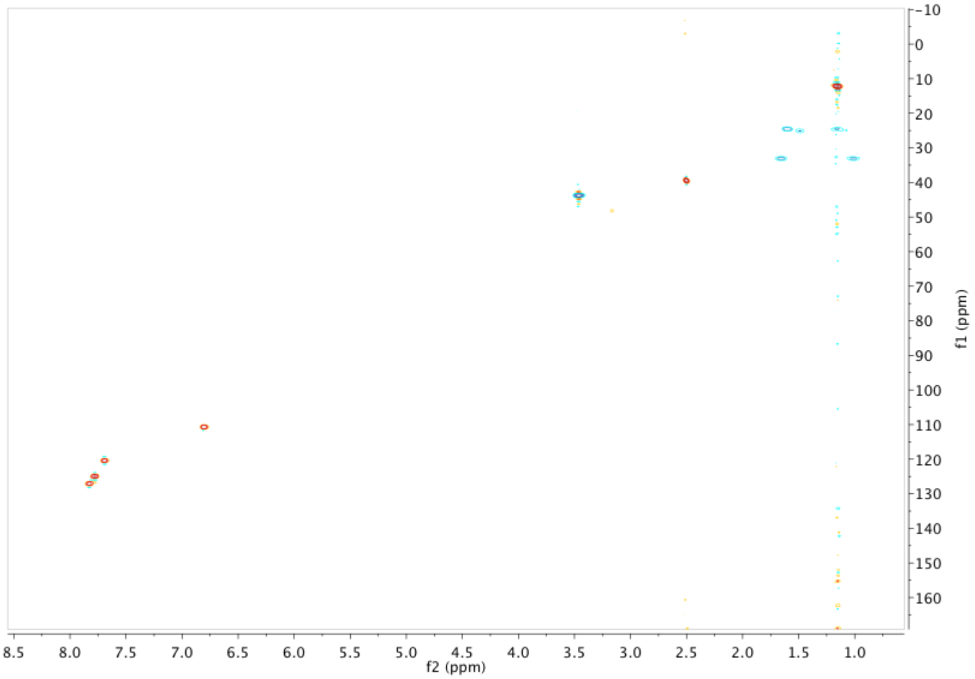

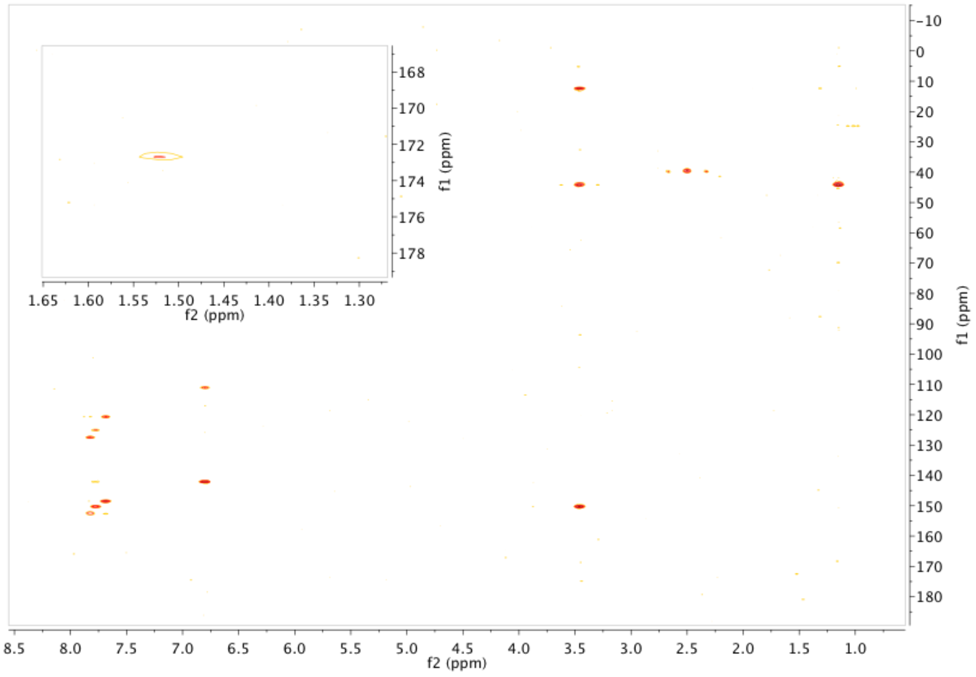

Supplementary Figure 4: 2D NMR of **JB253** (HSQC, top; HMBC, bottom).

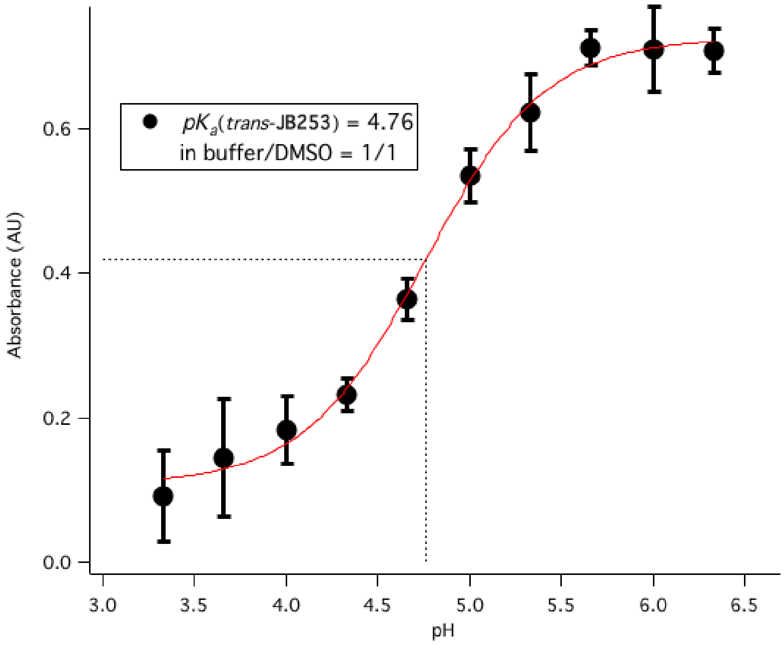

Supplementary Figure 5: *pK_a* measurement of JB253. Ionization constant of *trans*-**JB253** in a DMSO/buffer mixture (1/1) was determined with an absorbance-based platereader assay. All datapoints were acquired in triplicate (n = 3 repeats) at the following pH-values: 3.33, 3.66, 4.00, 4.33, 4.66, 5.00, 5.33, 5.66, 6.00 and 6.33. Ionic strength was held constant (I = 0.1 mM) by the addition of KCl to each buffer. Data was background subtracted and the spectral differences plotted against the pH. Sigmoidal fitting (IgorPro v6.22a) obtained a pK_a = 4.76. Values represent the mean ± SD.

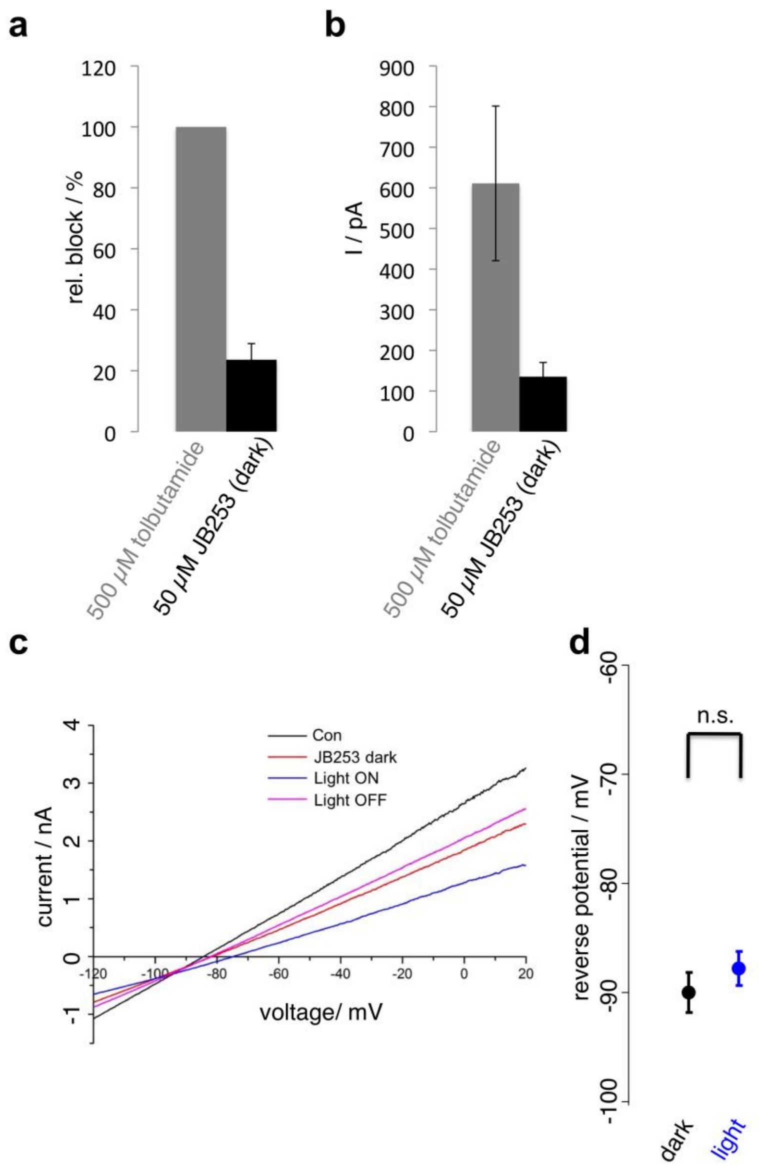

Supplementary Figure 6: K_{ATP} channel block characteristics of JB253 in the dark. **a)** To account for cell-cell variation in current densities, magnitude block with **JB253** (dark) was calculated as a percentage *versus* that achieved with 500 µM tolbutamide in the same experiment (*n* = 3 recordings). **b)** Bar graph displaying the amplitude of the inward current (ΔI [pA]) at -60 mV elicited by application of either tolbutamide or JB253 in the dark (*n* = 3 recordings). **c)** Representative current-voltage (IV) relationships showing a minor decrease in membrane conductance upon application of **JB253** in the dark (before drug = black; after drug = orange). This inhibition is reversibly enhanced by exposure to blue light (blue = ON; pink = OFF). **d)** Mean reversal potential (E_{rev}) before and after illumination of **JB253** measured in recordings as depicted in **c)**. (NS, non-significant, *trans*-**JB253** *versus* *cis*-**JB253**; Student's paired t-test) (*n* = 5 recordings). Values represent the mean ± SEM.

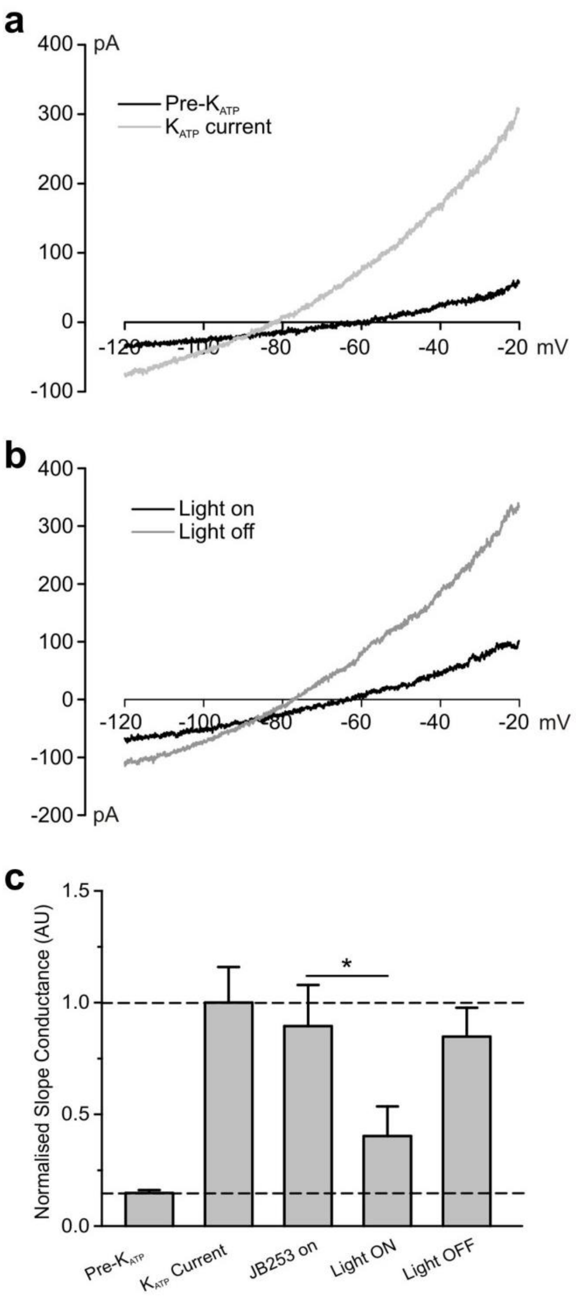

Supplementary Figure 7: JB253 reversibly blocks K_{ATP} currents in MIN6 beta cells. **a)** Representative current-voltage (IV) relationships recorded straight after establishing the whole-cell configuration (Pre-K_{ATP}) and after development of the K_{ATP} current due to wash-out of ATP from the cell (Peak K_{ATP} current). **b)** IVs from the same cell as a) in the presence of either *trans*-**JB253** (light off) or *cis*-**JB253** (light on). **c)** Bar graph displaying mean data from experiments as shown in **a)** and **b)**. Slope conductance was normalized to peak K_{ATP} current. Note that at this concentration (10 mM), **JB253** only blocks the K_{ATP} current during illumination and this is readily reversed when the light source is shut off (*$P<0.05$ *versus* **JB253** dark; one-way ANOVA) ($n = 4$ recordings). Values represent the mean ± SEM.

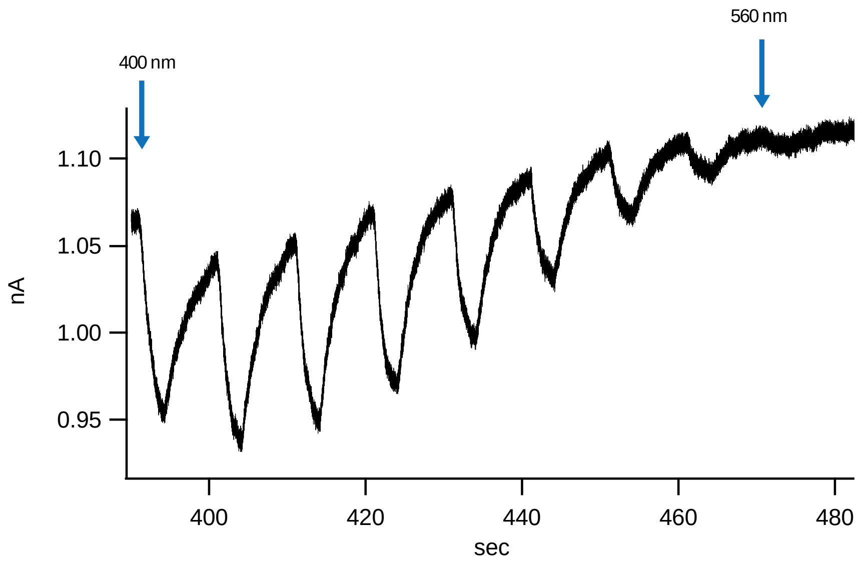

Supplementary Figure 8: Extended JB253 action spectrum. JB253 is unable to photoswitch K_{ATP} currents in HEK293t cells transfected with Kir6.2 and SUR1 at wavelengths > 560 nm (holding potential -60 mV) (trace representative of *n* = 3 recordings).

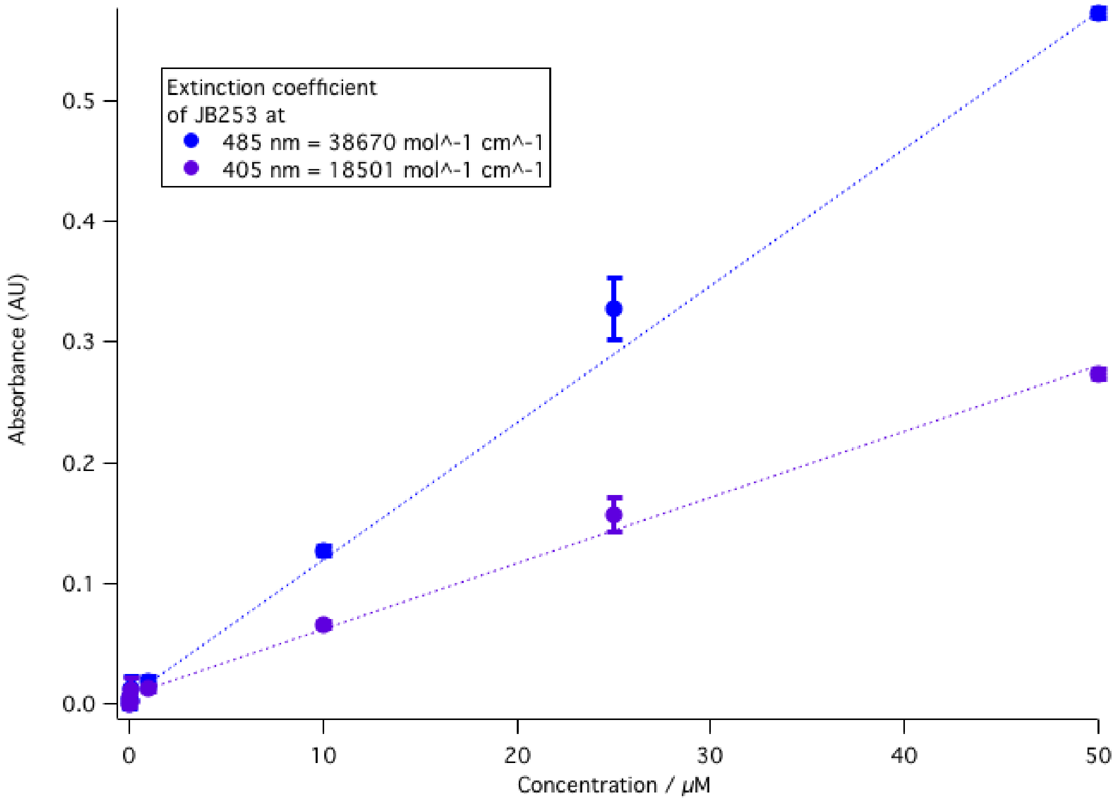

Supplementary Figure 9: Extinction coefficient measurement of JB253. Absorbance at two wavelengths (405 and 485 nm) was measured of a dilution series of **JB253** (concentrations in µM: 0.01, 0.10, 1.00, 10.0, 25.0 50.0) in low K^+ buffer (containing in mM: 3 KCl, 118 NaCl, 25 $NaHCO_3$, 2 $CaCl_2$, 1 $MgCl_2$, 10 HEPES, NaOH to pH 7.4) (*n* = 4 repeats). Data was background subtracted and the absorbance values plotted against the concentration to obtain the extinction coefficients *via* linear fitting. Values represent mean ± SD.

SUPPLEMENTARY TABLES

Supplementary Table 1: Crystallographic data for JB253

	**JB253**
net formula	$C_{23}H_{31}N_5O_3S$
M_r/g mol^{-1}	457.590
crystal size/mm	0.100 × 0.080 × 0.050
T/K	173(2)
radiation	'Mo Kα
diffractometer	'Bruker D8Venture'
crystal system	monoclinic
space group	$P2_1/n$
a/Å	15.8069(6)
b/Å	9.0578(3)
c/Å	17.1444(7)
α/°	90
β/°	95.8298(13)
γ/°	90
V/Å^3	2441.97(16)
Z	4
calc. density/g cm^{-3}	1.24466(8)
μ/mm^{-1}	0.166
absorption correction	multi-scan
transmission factor range	0.9045–0.9585
refls. measured	26927
R_{int}	0.0416
mean σ(*I*)/*I*	0.0408
θ range	3.28–27.52
observed refls.	4046
x, *y* (weighting scheme)	0.0742, 2.2726
hydrogen refinement	mixed
refls in refinement	5578
parameters	299
restraints	0
$R(F_{obs})$	0.0601
$R_w(F^2)$	0.1664
S	1.049
shift/error$_{max}$	0.001
max electron density/e Å^{-3}	1.022
min electron density/e Å^{-3}	–0.402

Supplementary Table 2: Wavelength-dependent kinetics and current change in JB253-treated cells. On/off kinetics for various wavelengths are shown in ms ± SD (*n* = 3 recordings). Current change is expressed as percentage (% ± SD)-block *versus* that obtained with tolbutamide in the same experiment (*n* = 3 recordings). Current change (ΔI [pA]) for a single representative experiment is displayed. In all cases, cells were exposed to 500 µM tolbutamide before washout and application of 50 µM **JB253**.

λ [nm]	τ_{on} [ms]	τ_{off} [ms]	% block	ΔI [pA]
400	1176±385	2758±745	44.0±29.6	216
420	1309±438	2209±482	69.4±51.1	492
440	1246±421	2295±397	70.0±44.3	440
460	1181±447	1940±322	72.8±48.1	481
480	1163±434	2183±422	72.4±49.0	487
500	1244±462	2002±354	70.2±49.5	482

Supplementary Table 3: Kinetics and current change during a repetitive illumination cycle. On/off kinetics are shown as ms ± SEM and current change as pA ± SEM calculated following four dark/400 nm cycles. Values are from a single cell.

τ_{on} [ms]	τ_{off} [ms]	ΔI [pA]
477±26	1472±75	504.8±6.9

Supplementary Table 4: Current change for first and last switch for all experiments. The difference in current change (ΔI [pA]) between the first and last switch (400nm) is represented as a percentage (%).

Experiment #	ΔI [pA] first switch	ΔI [pA] last switch	Cycles	Difference (%)
1	136.5	89.5	12	34.4
2	438.8	423.8	9	3.4
3	200.0	161.0	5	19.5
4	521	510	4	2.1
